# Recent Developments in Pathological pH-Responsive Polymeric Nanobiosensors for Cancer Theranostics

**DOI:** 10.3389/fbioe.2020.601586

**Published:** 2020-11-19

**Authors:** E. K. Pramod Kumar, Wooram Um, Jae Hyung Park

**Affiliations:** ^1^School of Chemical Engineering, College of Engineering, Sungkyunkwan University, Suwon, South Korea; ^2^Biomedical Institute for Convergence at SKKU (BICS), Sungkyunkwan University, Suwon, South Korea

**Keywords:** pH-responsive nanotheranostics, magnetic resonance imaging, ultrasound imaging, photoacoustic imaging, fluorescence imaging, photodynamic therapy, sonodynamic therapy, chemodynamic therapy

## Abstract

Polymeric nanobiosensors (PNBS) that respond to tumor-related factors, including pH, have shown great potential for disease detection owing to their selectivity and sensitivity. PNBS can be converted into theranostic polymeric nanobiosensors (TPNBS) by incorporating therapeutic cargo, thereby enabling concomitant diagnoses and therapy of targeted diseases. The polymeric compartments in TPNBS play a significant role in the development and therapeutic efficacy of nanobiosensors. Polymers enhance the stability, biocompatibility, and selective or effective accumulation of nanobiosensors at desired pathological sites. The intrinsic pH sensitivity of either the polymers in TPNBS or the TPNBS themselves provides integrated potentialities such as cogent accumulation of TPNBS at the tumor, augmented tumor penetration, cellular uptake, and theranostic activation, including enhanced bioimaging signals and controlled release of therapeutics. In this review, we summarize recent developments in the design, preparation, and characterization of pH-responsive TPNBS and their ability to behave as efficient *in vivo* nanotheranostic agents in acidic cancer environments.

## Introduction

Cancer is a major threat to human health worldwide. According to recent statistics, in 2020, the United States is projected to have 1,806,590 new cancer cases and 606,520 cancer-related deaths (Siegel et al., 2020). The cancer death rate increased until 1991 and then decreased continuously till 2017, resulting in an overall decrease of 29%. This was mainly attributed to the research and development in the field of cancer theranostics. Researchers have devoted significant efforts toward understanding the pathogenesis and properties of cancer in order to develop effective treatments for clinical applications. Compared to healthy tissues, the tumor microenvironment (TME), which is composed of cancer-stromal-immune cells and the extracellular matrix, is characterized by uncontrolled cell proliferation. This high cell growth rate is associated with a modified metabolism and the abnormal development of tumor blood vessels. This results in a reduction in the transport of oxygen and nutrients (Vaupel and Harrison, 2004). Approaches for identifying such characteristics of cancer cells have contributed considerably toward the development of tumor-specific nanotheranostics.

Oxidative phosphorylation is a vital pathway of normal cell metabolism; it releases chemical energy from molecular oxygen bonds through the enzymatic oxidation of nutrients during adenosine triphosphate (ATP) synthesis (Da Poian et al., 2010). Oxygen and nutrient deficiency in tumor tissues urge cells to produce energy for survival through anaerobic glycolysis, which differs from oxidative phosphorylation (Vander Heiden et al., 2009). Warburg et al. (1927) studied the anaerobic glycolysis of glucose in tumor veins and arteries. They found more lactic acid and less glucose in tumor tissues than on the arteries, which served as evidence of the modified metabolism in tumor cells. In addition to lactic acid, tumor cells release chemical energy stored in the phosphoanhydride bonds of ATP via hydrolysis, along with the release of carbon dioxide and protons. Thus, the anaerobic glycolysis, or “Warburg effect,” increases the acidity and lowers the pH of pathological sites compared to that in normal tissues. The extracellular pH values of healthy tissues and the late endosomes–lysosomes of normal cells are approximately 7.4 and 5–6.5, respectively, whereas those of the tumor tissue and the late endosomes–lysosomes of tumor cells are 6–7 and 4.5–6.5, respectively (Thews et al., 2006; Swietach et al., 2007; Kumar et al., 2014). These pH values may vary depending on the tumor; however, they are always lower than those of healthy tissues (Danhier et al., 2010). Given the acidic nature of the TME and the late endosomes–lysosomes of the cells, pH-sensitive cancer imaging and therapy using suitable theranostic nanomedicine agents have been developed (Webb et al., 2011).

Theranostic nanomedicine for cancer primarily involves the use of colloidal nanoparticles (NPs) ranging from 10 to 1000 nm in size (Muthu et al., 2014). Synthetic and natural polymers are vital constituents of these NPs, within which diagnostic and therapeutic agents (organic or inorganic theranostic agents) are encapsulated, conjugated, absorbed, or entrapped. The resulting theranostic polymeric nanobiosensors (TPNBS) simultaneously diagnose and treat cancer at the cellular level (Peng et al., 2015; Zhao et al., 2016). The polymer components improve aqueous stability and the delivery of theranostic cargoes associated with TPNBS (Kakkar et al., 2017; Kocak et al., 2017). An intravenously administered TPNBS may be sequestered by the reticuloendothelial-system (RES)-rich organs such as the liver and kidney. Research on theranostic nanomedicine allows for the development of TPNBS with a non-immunogenic polymer surface to increase their blood circulation half-life (Deepagan et al., 2013; Schöttler et al., 2016); this, in turn, improves their effective accumulation in leaky vasculature-containing tumors, which are associated with uncontrolled cell proliferation (the EPR effect) (Matsumura and Maeda, 1986). In addition to the passive accumulation of TPNBS in the tumor, resulting from the EPR effect, decorating its surface with tumor-specific targeting ligands, such as peptides, small molecules, antibodies, and aptamers, could further improve its targeted (active) accumulation through ligand–receptor interactions (Danhier et al., 2010; Rosenblum et al., 2018). However, the amount of administered TPNBS that can reach the tumor either via passive or active accumulation is still minimal (Wilhelm et al., 2016). This causes imaging and therapeutic functions to appear throughout the body rather than in the tumor alone, resulting in a poor signal-to-noise (S/N) ratio for imaging and an increased risk of systemic toxicity. Recent studies have focused on overcoming these issues by developing smart, stimuli-responsive TPNBS, which activate imaging and therapeutic functions only in response to endogenous stimuli (e.g., pH, enzymes, redox [glutathione (GSH)-reactive oxygen species (ROS), and hypoxia] or exogenous stimuli (e.g., ultrasound, light, microwave, radiofrequency, and magnetism) present or applied at targeted tumor sites (Feng Q.H. et al., 2018; Kang et al., 2018). Stimuli-responsive TPNBS are switched “on” only at the tumor tissues and afford the selective and sensitive enhancement of imaging and therapeutic efficacy. In contrast, they remain in the “off” state in normal tissues. These TPNBS with improved imaging and therapeutic efficiency provide valuable information, such as that required for detecting diseases, monitoring disease progression, and evaluating patient response to therapy, by performing simultaneous imaging and treatment.

Stimuli-sensitive nanotheranostics offer advantages over conventional passive or active tumor-targeting strategies. Therefore, different stimuli-responsive nanotheranostic agents have been developed (Alsehli, 2020; Gong et al., 2020; Mi, 2020; Parodi et al., 2020). pH-responsive nanotheranostic agents have gained popularity owing to their ability to activate theranostic functions in response to the pH difference among the normal cells, TME, and intracellular microenvironment of tumors. A large number of polymeric and polymer-coated inorganic nanomaterials exhibiting different physicochemical properties in response to changes in the pH have been employed for the preparation of acidic TMEs and intracellular microenvironment-responsive smart nanotheranostics (Feng Q.H. et al., 2018). pH-sensitive hydrophobic to hydrophilic transitional polymers featuring protonatable groups (Bazban-Shotorbani et al., 2017; Wu et al., 2018), pH-sensitive cleavable linkers containing polymers (Kanamala et al., 2016; Wu et al., 2018), pH-responsive charge and size convertible polymers (Yang et al., 2018; Dai et al., 2020), and pH-sensitive inorganic core-forming materials (Dong et al., 2016), among others, are being used to fabricate nanotheranostic agents (Table 1).

**TABLE 1:** Summary of pH-responsive nanotheranostics.

Operational pH	Mechanism of pH sensitivity	pH-responsive chemical structure	Functions	References
∼6.8	Cyclization-amide bond degradation (Kang et al., 2014)	DMMA-PEG	(1) Charge reversal (2) PDT, Immunotherapy	Yang et al., 2018; Peng et al., 2020
∼6.8	Imine bonds	Benzoic-imine	(1) Charge reversal (2) PTT	Lei et al., 2017
∼6.8	Boronate ester	PBA-Gal	(1) Ligand-reversible shielding (2) PDT	Cao et al., 2019
∼6.0	Boronate ester	PBA-DA	(1) Charge reversal and size reduction (2) PTT, PDT, Chemo, PAI, FI	Cong et al., 2020
∼6.8	Cyclization-amide bond degradation	DMMA-PEG	(1) Charge reverse and size reduction (2) Chemo and immunotherapy	Dai et al., 2020
∼6.8	Peptide folding	Melittin peptide	(1) Morphology switching (2) PTT	Jia et al., 2019
∼5.0–6.7	CaP degradation	CaP	(1) Contrast agent (*T*_1_) release (2) MRI	Mi et al., 2016
∼5.5–7.0	Structural transformation	i-Motif DNAs	(1) Contrast agent conversion (*T*_2_ to *T*_1_) (2) MRI	Lu et al., 2018
∼5.0	Pore formation	FA-FRT-PFP	(1) Droplet vaporization (2) USI	Li et al., 2020
∼5.5	Protonation	pH-BDP	(1) Ratiometric PAI of pH	Miao et al., 2016
∼ < 7.0	Protonation	BSA–PANI	(1) Amplified PAI	Tian et al., 2019
∼6.8	FRET	ANNA-Cy_5.5_	(1) Dual ratiometric FI of pH	Ma et al., 2018
∼4.5–5.0	Porosity, protonation	NEt_2_Br_2_BDP	(1) Amplified FI	Tian et al., 2015
∼6.0–6.2	Protonation	mPEG-*b*-PDPA-Cy_7.5_	(1) Augmented PDT and FI	Qi et al., 2019; Yang et al., 2019
∼6.0	Cyclization-amide bond degradation	DEX-MMfu	(1) Enhanced permeability of TME (2) PAI, PDT, and Immunotherapy	Wang et al., 2019
∼4.3–5.8	Degradation	CaCO_3_	(1) Bursting effect (2) USI, SDT	Feng Q. et al., 2018
∼6.8	Degradation	MnO_2_	(1) O_2_ release (2) MRI, SDT	Lin et al., 2020
∼6.8	Degradation	MnS	(1) MRI and CDT agent release (2) H_2_S release	He et al., 2020
∼4.4–5.4	Degradation	MnO_x_	(1) New mechanism of CDT (2) FI	Lu et al., 2020

Conventional cancer treatment methods such as chemotherapy, surgery, and radiotherapy fail to cure cancer completely as they are associated with the impairment of the host immune system, adverse drug reactions, poor patient adherence, and low therapeutic efficiency (Nowak et al., 2002; Couzin, 2008; Chaffer and Weinberg, 2011). Thus far, several pH-responsive smart nanomaterials have been developed to overcome these limitations for chemotherapy. Additionally, several studies have convincingly summarized pH-sensitive drug delivery (Mura et al., 2013; Kanamala et al., 2016; Wang et al., 2018; Deirram et al., 2019). The development of minimally invasive or non-invasive therapeutic modalities [such as light, ultrasound (US), and endogenous ROS] and induced therapies [such as photothermal-photodynamic therapy (PTT-PDT) (Jaque et al., 2014; Lucky et al., 2015), sonodynamic therapy (SDT) (You et al., 2016), and chemodynamic therapy (CDT) (Tang et al., 2019)] are currently being used in theranostic nanomedicine for cancer treatment. These treatment protocols only damage cancer cells, while leaving normal cells unaffected and intact. The non-invasive therapeutic efficacy can be improved by developing pH-responsive nanotherapeutic agents.

In recent years, pH-responsive PNBS offering magnetic resonance imaging (MRI), photoacoustic imaging (PAI) fluorescence imaging (FI), ultrasound imaging (USI), and capabilities of improved signal-to-noise ratios in tumors have been developed (Kumar et al., 2013; Kaittanis et al., 2014; Ling et al., 2014; Søndergaard et al., 2015; Wang X. et al., 2015; Wang et al., 2017; Mi et al., 2016; Miao et al., 2016; Zhang et al., 2017b; Vidallon et al., 2020). Therapeutic cargo-incorporated PNBS (TPNBS), which are capable of simultaneous pH-responsive imaging and therapy, have also attracted significant research attention (Wang et al., 2013; Li et al., 2017; Feng Q.H. et al., 2018; Zhu et al., 2018; He et al., 2020). In this review, we evaluate how the development of pH-responsive multifunctional nanomaterials has contributed toward improvements in cancer treatment using the capabilities of these sensors to increase tumor accumulation, tumor penetration, cellular uptake, and theranostic functions. We summarize important recent developments in the design and fabrication of (1) pH-responsive charge and size convertible polymeric nanomaterials for augmented non-invasive cancer therapy, (2) pH-sensitive PNBS for disease detection and bioimaging, and (3) pH-responsive TPNBS for image-guided non-invasive cancer therapy.

## pH-Responsive Charge and Size Convertible NPs for Augmented Non-Invasive Cancer Therapy

The treatment outcomes of theranostic nanomedicine primarily depends on prolonged *in vivo* blood circulation, intratumoral penetration, and the subsequent tumor cell internalization of the NPs (Alexis et al., 2008). Rapid clearance of circulating NPs during systemic delivery via RES is a critical issue in nanomedicine. Therefore, it is necessary to minimize the factors that reduce the blood circulation half-life and biodistribution of NPs while designing for theranostic applications. The NP–RES interactions are influenced by factors such as NP composition, size, surface charge, stability, and surface modifications (pegylation and targeting ligands). pH-responsive charge and size-tunable theranostic NPs have been developed to minimize interactions, thereby improving therapeutic efficacy in the tumor. These NPs enhance blood circulation time, selective tumor accumulation, penetration, and cellular internalizations.

### pH-Responsive Charge Convertible NPs

It is well known that NPs with neutral or negatively charged surfaces prevent RES clearance and lengthen the blood circulation half-life, leading to effective accumulation in tumor tissues owing to the EPR effect. However, a positive surface charge on the NPs would rapidly clear them from the blood through the RES (Yuan et al., 2012; Feng et al., 2014). This positive surface charge, however, facilitates intra-tumoral penetration and the ensuing tumor cell internalization of the NPs (Cho et al., 2009). Therefore, NPs with swappable surface charges that are neutral or negative during blood circulation and quickly convert to positive charges on reaching the acidic TME have recently been annotated as a promising strategy for pH-responsive augmentation in therapy (Han et al., 2016; Zhang et al., 2020).

**FIGURE 1:**
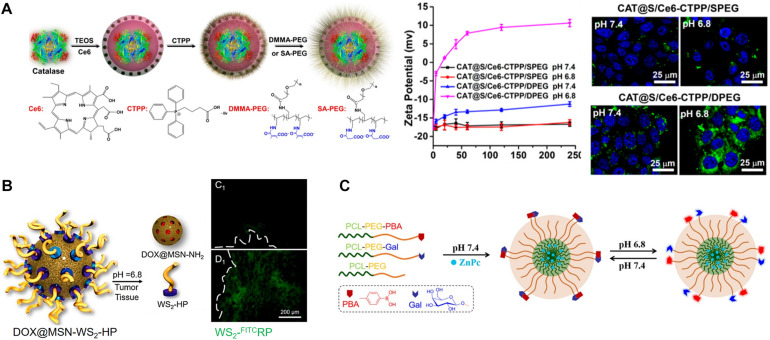
**(A)** TME pH-responsive charge reversing NPs for enhanced intracellular mitochondrial targeting and improved PDT. Reproduced with permission from reference (Yang et al., 2018). Copyright 2018, American Chemical Society. **(B)** TME pH-responsive degradation cluster bomb and enhanced tumor penetration of the released WS_2_−^FITC^HP quantum dots compared to its control (WS_2_−^FITC^RP), confirmed via *ex vivo* imaging (the dotted line represents the edge of tumor sections). Reproduced with permission from reference (Lei et al., 2017). Copyright 2017, American Chemical Society. **(C)** pH-responsive ligand-reversible-shielding micelle for enhanced cellular uptake with the associated augmented PDT. Reproduced with permission from reference (Cao et al., 2019). Copyright 2019, American Chemical Society.

The non-immunogenic polymer, such as polyethylene glycol (PEG), shielding on NPs creates an inert NP surface that enhances their blood circulation half-life and passive tumor accumulation (Fam et al., 2020). Although the dense PEG corona on NPs affords improved tumor accumulation, the PEGylated NPs exhibit reduced intratumoral penetration, succeeding tumor cell internalization, and endosomal escape (Mishra et al., 2004; Kong et al., 2019). Therefore, significant research efforts have devoted toward designing TME pH-responsive PEG corona deshielding NPs; the removal of the PEG corona converts surface charges into positive charges, thereby improving the intratumoral penetration and tumor cell internalization of these NPs. Yang et al. (2018) designed a pH-responsive PEG corona with smart nanoreactors for mitochondrial targeting and enhanced photodynamic-immuno combination therapy. Water-soluble, H_2_O_2_-decomposing, and oxygen-producing enzyme catalase (CAT)-encapsulated and photosensitizer (Ce6)-loaded hollow silica NPs (S) were prepared (Figure 1A). The NP surface was modified using the mitochondria-targeting molecule 3-carboxypropyl-triphenylphosphonium bromide (CTPP), and the resulting cationic surface was further modified with a pH-responsive charge convertible corona forming anionic PEG 2,3-dimethylmaleic amide (DMMA) polymer (PEG-DMMA) via electrostatic interaction. The obtained CAT@S/Ce6-CTPP/D-PEG featured a well-defined hollow structure with a negative surface charge at neutral pH because of the PEG shielding. After effective accumulation of the long-circulating NPs in the TME, due to the TME acidity, PEG was detached from the NPs owing to the pH-sensitive cyclization-induced degradation of the PEG-DMMA amide linkage. This detachment results in a CTPP-exposed cationic NP surface, enabling the effective intracellular targeting of mitochondria, thereby augmenting PDT-induced cancer cell destruction, in comparison with control NPs (CAT@S/Ce6-CTPP/S-PEG) with pH-inert PEG-succinic anhydride (SA-PEG). In the intracellular microenvironment, the catalase encapsulated within these NPs acts as a nanoreactor to trigger the decomposition of tumor-rich endogenous H_2_O_2_, production of O_2_ used to relieve tumor hypoxia, and further augmentation of *in vivo* PDT efficacy. A combination of augmented PDT with checkpoint blockade immunotherapy and programmed death-ligand 1 (PD-L1) antibody significantly boosted the infiltration of cytotoxic T lymphocytes (CTLs) into remote tumors. It impeded their growth, demonstrating a robust abscopal effect encouraging metastasis inhibition. Similarly, pH-responsive charge reversing NPs featuring enhanced tumor targetability and mitochondrial accumulation for PD-immunotherapy were reported recently (Peng et al., 2020). Sequential-targeting positively charged micelles with a Ce6 loaded core were prepared from amphiphilic poly (beta-amino ester), composed of hydrophilic CTTP-PEG and hydrophobic thioketal polymer blocks. The micelles were coated with an anionic pH-responsive charge-transforming shell-forming polymer with tumor-targeting capability (BiotinPEG_4000_-DMMA) using electrostatic interaction. Additionally, pH-responsive charge conversion was used to augment mitochondrial targeting, thereby improving PDT efficacy.

Lei et al. (2017) prepared a TME pH-responsive “Cluster Bomb” composed of a drug (DOX) containing loaded mesoporous silica NPs (MSN) capped with tumor-homing or tumor-penetrating peptide tLyP-1-modified tungsten disulfide quantum dots (WS_2_-HP), by using pH-sensitive benzoic-imine bonds for programmed tumor therapy. The prepared DOX@MSN-WS_2_-HP with the surface-decorated peptide tLyP-1 promotes the tumor-homing ability of 4T1 (Figure 1B). The benzoic-imine bonds between MSN and the quantum dots remain stable during blood circulation. However, they become highly unstable when the TME pH is 6.8. Once the cluster bomb is accumulated in the TME, it breaks into two parts: DOX@MSN-NH_2_ and tLyP-1weraing WS_2_-HP. Charge-converted electropositive DOX@MSN-NH_2_ enabled efficient chemotherapy on surface tumor cells. The deep tumor-penetrating WS_2_-HP showed tumor suppression in the deep-seated tumor cells by near-infrared (NIR)-light-triggered PTT.

The Yuan group recently reported another interesting strategy of pH-sensitive “ligand-reversible-shielding micelle” for increased tumor accumulation associated with effective PDT (Cao et al., 2019). Phenylboronic acid-functionalized poly(ethylene glycol)-*b*-poly(ε-caprolactone) (PBA-PEG-PCL), and galactose-functionalized diblock polymer (Gal-PEG-PCL) were mixed to prepare dual-ligand micelles (PBA/Gal) to demonstrate this concept (Figure 1C). The PBA and Gal residues form boronate ester complexes at a pH of 7.4, which mutually shield their targeting function. In an acidic TME (pH 6.8), the binding affinity between the PBA and Gal is weakened, and the PBA prefers to bind with the sialic acid residues expressed on the tumor cell surface rather than the Gal on the micellar surface. Furthermore, the unbound Gal recovers its targeting ability toward the asialoglycoprotein receptor, leading to an enhancement in cellular uptake through receptor-mediated endocytosis. A photosensitizer, zinc phthalocyanine (ZnPc), was loaded onto the reversible-shielding micelle to augment the PDT, owing to its pH-responsive enhanced cellular uptake.

### pH-Responsive Size Convertible NPs

It has been reported that NPs with a size of 10–100 nm and suitable surface modifications achieve efficient tumor accumulation during systemic administration via the EPR effect. NPs smaller than 10 nm have shown rapid clearance via renal excretion, resulting in reduced tumor accumulation (Cabral et al., 2011; Blanco et al., 2015; Hoshyar et al., 2016). However, compared to large NPs (10 nm<), small NPs (10 nm>) have shown improved capabilities of intratumoral penetration and subsequent tumor cell internalization (Blanco et al., 2015). Therefore, to solve the size-related conflict issues of NPs, TME or intracellular pH-responsive size swappable NPs have been developed (Sun et al., 2014; Wang J. et al., 2015). In acidic tumors, large NPs accumulated via the EPR effect could undergo a pH-responsive size shrinkage or degradation into smaller NPs. This size reduction improves tumor penetration and cell internalization, resulting in augmented therapeutic efficacy (Li et al., 2015; Li H.-J. et al., 2016).

**FIGURE 2:**
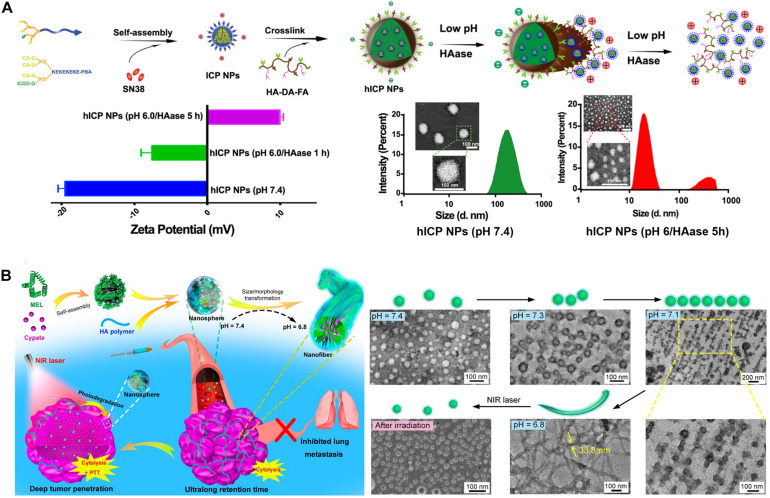
**(A)** pH-responsive charge and size convertible hICP NPs. Reproduced with permission from reference (Cong et al., 2020). Copyright 2020, American Chemical Society. **(B)** pH-responsive morphological transformation of MEL/Cypate@HA nanospheres followed by their photodegradation into small nanospheres. Reproduced with permission from reference (Jia et al., 2019). Copyright 2019, American Chemical Society.

Cong et al. (2020) reported hyaluronan (HA), stacked self-assembled NPs with folic acid (FA) and HA-mediated active targeting capabilities, upon pH-responsive intracellular transformations of size and surface charge. These transformations simultaneously augment tumor penetration, internalization, and intracellular drug release, as well as synergistic photo-chemo combination therapy. They prepared an amphiphilic hexadecapeptide decorated with three cholic acids (CA), one indocyanine green derivative (ICGD), and one 4-carboxy-3-fluorophenylboronic acid (PBA) (Figure 2A). This amphiphilic polypeptide was self-assembled into positively charged small and stable micelle NPs (<30 nm) (ICP NPs) that are loaded with the antineoplastic drug (SN-38) with a critical micelle concentration (CMC) value of 0.04 μM, indicating high thermodynamic stability. The ICP NPs were then stacked using dopamine (DA), and folic acid (FA) decorated with HA (HA-DA-FA). This resulted in the formation of large (130 nm), negatively charged hICP NPs formed through the development of pH-responsive boronate ester bonds between the DA of the HA-DA-FA and the PBA surface wearing the ICP NPs. Additionally, electrostatic interactions between the negatively charged HA and the positively charged ICP NP surface further strengthens NP stacking. The non-immunogenic HA surface provides improved blood circulation time for the hICP NPs and subsequently accumulates at the TME of subcutaneous B16 melanoma-bearing mice. The hICP NPs were taken up by cancer cells via HA- and FA-mediated endocytosis. At the intracellular level, the hICP NPs underwent size and surface charge transformations in the presence of the enzyme hyaluronidase (HAase) in an acidic pH in the endosomes/lysosomes; they released small ICP NPs and accelerated intracellular drug release. The transformed ICP NPs facilitated transcytosis-mediated penetration within the tumors. The ICGD in the ICP NPs not only provides NIR light-induced PDT-PTT but also photoacoustic and NIR imaging capabilities. Furthermore, synergistic augmentation of chemo-photo therapeutic efficacy was achieved owing to the pH-responsive size and charge transformation capabilities of the NPs.

Another type of pH-responsive size and charge transformable NPs was prepared and programmed by overcoming the delivery barrier for improved chemo-ido immunotherapy (Dai et al., 2020). The pH-responsive detachment of DMMA-PEG from a prodrug micelle converts its negative charge of −5.2 mV to a strongly positive charge of 20 mV and reduces its size from 85 nm to 42 nm. The transformed micelles, with reduced size and positive surface charge, enhance tumor penetration and improve endocytosis efficiency; they also synergistically inhibit melanoma growth through the combined effects of chemotherapy-induced antitumor immune response and IDO-blockade immunotherapy. The pH-responsive morphological switching of peptide-cyanine nanostructures, self-assembled by peptide melittin (MEL), and a NIR-absorbing photothermal cyanine dye, Cypate, which is further coated with an HA polymer for tumor targeting (termed MEL/Cypate@HA), has recently been reported (Figure 2B) (Jia et al., 2019). In an acidic TME, the MEL/Cypate@HA nanosphere, which transforms into net-like nanofibers, affords an inhibitory effect on tumor cell mobility and also increases its tumor retention time. The pH-responsive peptide sequence in MEL was found to be responsible for the morphological transformation. The nanofibers were photodegraded into small nanospheres (25 nm) via NIR laser irradiation during the cypate-mediated PTT, which further improved deep tumor penetration of the loaded MEL and the MEL-induced cytolysis.

## pH-Responsive PNBS for Tumor Imaging

Imaging of heterogeneous tumors is essential for early detection, diagnoses, and precise treatment monitoring. The unique acidic characteristic of tumors enables the design of PNBS with a pH-responsive amplification of the signal-to-noise (S/N) ratio during tumor-specific imaging (Feng L. et al., 2018; Yang et al., 2020). Thus far, numerous pH-responsive smart PNBS have been reported for *in vivo* cancer imaging using techniques such as MRI, PAI, FI, and USI (Li Y. et al., 2016; Liu et al., 2017). In this section, we describe recent reports of pH-responsive imaging signal amplification in tumors and ratiometric imaging of pH in tumors.

### pH-Responsive MRI

Non-invasive imaging techniques, such as MRI, provide a high spatial resolution and detailed three-dimensional anatomical images (Kircher and Willmann, 2012). During MRI, the magnetic field introduced by powerful magnets forces protons in the body to align with the magnetic field. A radiofrequency current is then pulsed through the patient, which stimulates the protons to spin out of equilibrium, causing strain against the pull of the magnetic field. Once the radiofrequency field is switched off, the protons relax back or realign with the magnetic field by releasing energy, which is detected using MRI sensors. The proton relaxation time, as well as the energy released during this process, depends on the chemical nature of the protons and their environments. Longitudinal or spin-lattice relaxation time (*T*_1_) and transversal (*T*_2_) or spin-spin relaxation times of protons are involved in MRI, where *T_2_* ≤ *T*_1_. The relaxation times (*T*_1_ or *T*_2_) of water protons in lesions can be sped up using an MRI contrast agent. The *T*_1_ and *T*_2_ contrast agents increase the S/N ratios or the contrast of the lesion for more accurate diagnoses of cancer-related pathological diseases. Therefore, the delivery of MR contrast agents to the tumor by using tumor acidity to improve the S/N of imaging has received significant research attention.

**FIGURE 3:**
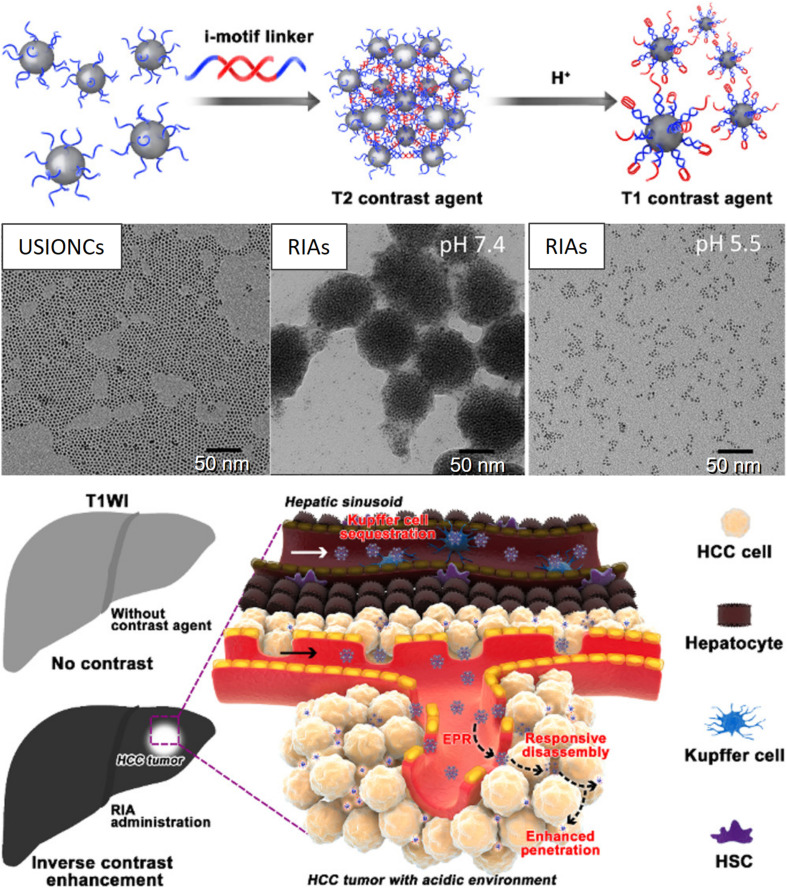
pH-responsive conversion of the MRI contrast agent confirmed via TEM images, and used for inverse contrast enhancement for the detection of small hepatocellular carcinomas (HCCs). Reproduced with permission from reference (Lu et al., 2018). Copyright 2018, American Chemical Society.

For example, a *T*_1_ (longitudinal) MR contrast agent, releasing PNBS composed of MnO_2_, has been extensively investigated for its ability to release Mn^2+^ ions in acidic tumors (Cai et al., 2019). Mi et al. (2016) prepared Mn^2+^-doped calcium phosphate (CaP) NPs for the pH-responsive MRI contrast on-off system for signal amplification in subcutaneous C26 tumor-bearing mice. CaP NPs were prepared via the mineralization of poly(ethylene glycol)-*b*-poly(glutamic acid) [PEG-b-P(Glu)] block copolymers with Mn^2+^, Ca^2+^, and HPO${}_{42}^{-}$ in the aqueous phase, followed by hydrothermal treatment to improve the mechanical strength of the CaP matrix. The PEG shell in the resulting PEGMnCaP helped maintain the size of the NPs at ∼60 nm by preventing further expansion of the CaP core. The CaP core of accumulated PEGMnCaP NPs was dissolved in the acidic TME, thereby releasing Mn^2+^ ions. This Mn^2+^ readily interacted with surrounding proteins and slowly rotated the Mn-protein system, which enhanced the sharp contrast due to the increased *T*_1_ relaxivity of Mn^2+^. Additionally, the PEGMnCaP NPs rapidly and selectively brightened and detected hypoxic regions in solid tumors and detected invisible millimeter-sized metastatic tumors in the liver, owing to their pH-responsive signal amplification capabilities. According to another report, a *T*_2_ contrast agent, such as Fe_3_O_4_ NPs, was loaded in the hydrophobic core of a pH-sensitive polymer (PEG-poly(β-amino ester) (PAE) micelle and was released and exposed to water molecules at the acidic tumor. Furthermore, a shortened water proton relaxation time *T*_2_ responsible for contrast enhancement during MRI was reported (Gao et al., 2010). Recently, a pH-responsive conversion of i-motif DNA-assisted iron oxide nanocluster assemblies (RIAs) from a *T*_2_ to a *T*_1_ contrast agent for the detection of small hepatocellular carcinomas (HCCs) has been reported (Figure 3) (Lu et al., 2018). The i-Motif DNAs exhibit pH-responsive structural transformation from single-stranded to intercalated quadruple-helical structures. In the acidic TME, the *T*_2_ contrast agent RIAs (120 nm), composed of the *T*_1_ contrast agent ultrasmall iron oxide nanoclusters (USIONCs), is disassembled back to USIONCs. The pH-dependent detachment of the i-Motif cross-linker is responsible for this intelligent contrast agent transformation, resulting in bright HCCs and a healthy dark liver owing to the inverse contrast-enhancing imaging mode transformation.

### pH-Responsive USI

Ultrasound imaging is portable, cost-effective, and enables real-time imaging. Despite these advantages, the low contrast in USI decreases its sensitivity, thereby limiting its diagnostic applications. Thus, contrast-enhanced US (CEUS), which utilizes 1–10-μm microbubbles (MBs) as the contrast agent, has been developed (Sun and Emelianov, 2019). However, these MBs cannot accumulate at disease sites via the EPR effect owing to their large sizes, limiting their application *in vivo*. Thus, the size of contrast agents should be in the nanometer range in order to accumulate at disease sites. Therefore, reducing the size of MBs and transforming them into nanobubbles (NBs) appears as a straightforward solution. However, NBs have low stability and low echogenicity, and they are excessively small to efficiently scatter ultrasonic waves at the frequencies used in clinical applications. Gas-generating NPs are introduced as a contrast agent for US imaging to overcome these issues and generate sufficient USI contrast while maintaining the nanometer-scale size.

**FIGURE 4:**
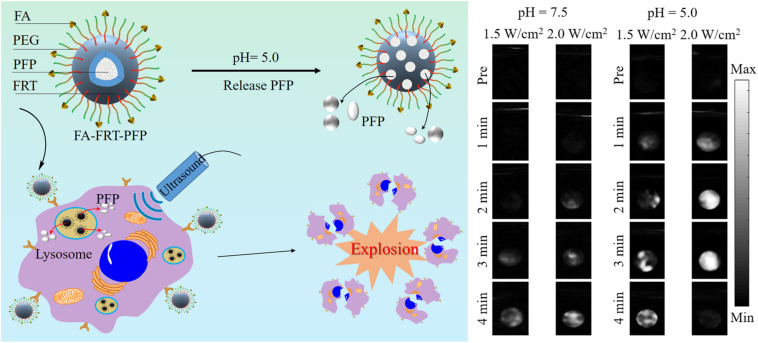
pH-responsive USI using PFP releasing FA-FRT-PFP nanoprobes, which shows enhanced USI contrast at acidic pH. Reproduced with permission from reference (Li et al., 2020). Copyright 2020, Springer.

pH-sensitive release of carbon dioxide (CO_2_) gas from mineralized calcium carbonate polymeric NPs (Min et al., 2015; Kim et al., 2018; Chen et al., 2019) or carbonate side chains containing polymer NPs (Kang et al., 2010) has been reported. These NPs exhibited strong echogenic signals with excellent echo persistence in tumoral acidic pH by producing CO_2_ bubbles. Recently, Li et al. (2020) prepared a US therapeutic agent that encapsulates low boiling (bp = 29°C) perfluoropentane (PFP) into pH-sensitive cage protein ferritin (FRT) conjugated with the tumor-targeting molecule FA (FA-FRT-PFP) (Li et al., 2020). FRT disassembles at intracellular pH and releases PFP. A 3-min, low-intensity focused ultrasound (LIFU, 2 W/cm^2^), which significantly enhances the US signal of the FA-FRT-PFP through the acoustic droplet vaporization (ADV) effect, is produced by the PFP (Figure 4). Additionally, the long duration (4 min) of LIFU irradiation at the lysosomal pH produced a physical shock wave due to the explosive release of PFP from the FA-FRT-PFP; this subsequently results in effective tumor cell destruction via necrosis. Another example of pH-responsive gas (O_2_) generation for USI is the use of manganese dioxide (MnO_2_) NPs that can aptly regulate TME oxygenation owing to their hydrogen peroxidase properties and favorable behavior of breaking-up in mildly acidic and H_2_O_2_-rich TMEs (Gao et al., 2017). The oxygen content in the tumor is elevated to 2.25 ± 0.07 times that in the absence of MnO_2_, which demonstrates its potential for USI.

### pH-Responsive PAI

Photoacoustic imaging is an emerging hybrid modality that combines optical excitation with ultrasonic detection. It allows for deeper tissue penetration and higher spatial resolution compared to traditional optical imaging techniques, owing to the involvement of deep tissue-penetrating ultrasound modalities (Miao and Pu, 2018). Endogenous (such as hemoglobin and melanin) and exogenous (such as NIR-emitting dyes, metallic NPs, carbon nanotubes, prophysomes, and 2D graphene analogs) PAI agents have been used for imaging applications. The PA signal depends on the difference in concentration between the healthy and diseased tissues. The poor accumulation and uptake of PAI agents could result in a poor S/N ratio in a complex pathological environment. Therefore, activatable PNBS that undergo intrinsic PAI signal amplification only in response to tumor-specific stimuli, including pH, and maintain a dormant state in the absence of pH can provide a high signal-to-noise ratio at the acidic tumor site. pH-sensitive activation provides a real-time correlation between PNBS states (activated vs. dormant) in pathological processes at the molecular level. This pH-responsive contrast enhancement has garnered significant research attention in recent years.

**FIGURE 5:**
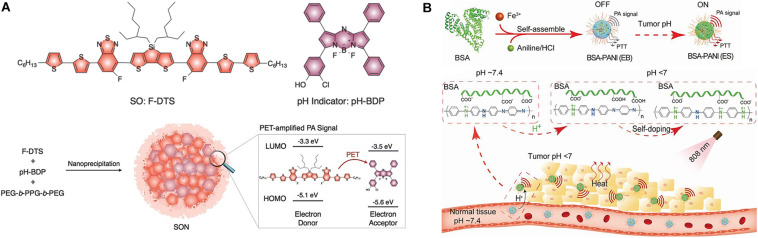
**(A)** Nanoprobe design of PET-amplified PA imaging of pH. Reproduced with permission from reference (Miao et al., 2016). Copyright 2016, Wiley-VCH GmbH. **(B)** pH-responsive amplification of PAI using BSA–PANI nanoprobe. Reproduced with permission from reference (Tian et al., 2019). Copyright 2019, Wiley-VCH GmbH.

Miao et al. (2016) developed activated ultrasmall semiconducting oligomer nanoprobes (SONs) with amplified signals for the *in vivo* PAI of pH. The SONs are prepared via nanoprecipitation of a low-molecular-weight semiconducting oligomer (SO:F-DTS) as the inert PA matrix, doped with a pH indicator dye, boron-dipyrromethene (pH-BDP), and served as a PA enhancer and amphiphilic triblock copolymer (PEG-*b*-PPG-*b*-PEG) as a coprecipitant (Figure 5A). The pH-BDP has a hydroxyl group on its backbone, which undergoes protonation upon acidification and imparts the pH-sensing ability of the SONs. Additionally, the electron affinity and ionization potential of the pH-BDP are lower than those of the SO:F-DTS. Thus, photoinduced electron transfer (PET) is favored between these binary optical partners, which results in the quenched fluorescence of SO:F-DTS; this, in turn, enhances the PA brightness of the SONs. Compared to the pH-inert non-doped SON (SON_0_), the PA brightness of the 50 wt% pH-BDP-doped nanoprobe (SON_50_) was substantially amplified by a factor of approximately 3.1 at 680 nm. Simultaneously, its ratiometric PA signal (PA_680_/PA_750_) increased by approximately 3.1 times on varying the pH from 7.4 to 5.5 *in vitro*. Systemic administration of SONs permits non-invasive real-time ratiometric PAI of the pH with the amplification of brightness in tumors of living mice. Another interesting example of pH-responsive PAI amplification was reported using a polyaniline (PANI)-based theranostic agent composed of bovine serum albumin (BSA) and PANI (Figure 5B) (Tian et al., 2019). The monomer aniline (ANI) polymerizes to PANI in the presence of BSA and Fe^3+^ ions, and the interaction between hydrophilic BSA and hydrophobic PANI self-assembles to BSA–PANI NPs. The strong BSA–PANI interaction leads to a pH-dependent self-doping effect through intermolecular acid-base reactions between the carboxyl groups of BSA and the imine moieties of the PANI backbone. This increases the degree of protonation of PANI and mediates its transformation from the emeraldine base (EB) to the emeraldine salt (ES) state in an acidic TME (pH < 7). Therefore, in healthy tissues (pH ∼7.4), the BSA–PANI assemblies exhibit low PAI signals and PTT effects during blood circulation, whereas they exhibited amplified PAI and enhanced PTT in acidic TMEs.

### pH-Responsive FI

Although FI is one of the most widely explored tumor imaging modalities, which also offers the advantages of ease of operation, high sensitivity, and multicolor imaging capabilities, its applications are limited owing to its low depth of light penetration in tissues and poor S/N ratio (Guo et al., 2014; Jin, 2019). The poor S/N in FI is attributed to the non-specific distribution of the imaging probe in tissues and background autofluorescence. pH-responsive fluorescence on-off PNBS are being developed to improve the S/N ratio, which can differentiate tumors from their surrounding normal tissue. The FI signal from PNBS is always turned off in the absence of tumor-specific stimuli (pH), providing pH-sensitive augmentation of FI at an acidic TME.

**FIGURE 6:**
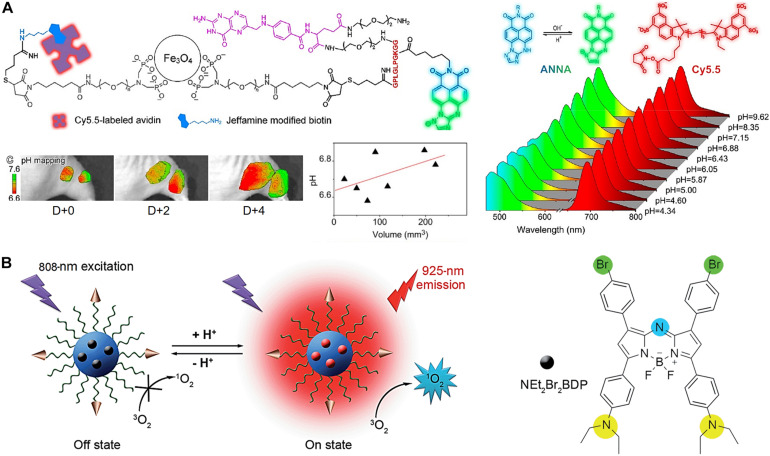
**(A)** Dual-ratiometric fluorescent nanoprobe for *in vivo* pH mapping. Protonation-/deprotonation-induced structural transformation of ANNA combined with pH-insensitive Cy_5_._5_ yields two sets of fluorescence spectra recorded at different pH values excited at 455 and 675 nm, providing ratiometric pH monitoring capabilities. The intratumorally injected nanoprobes allow for TME pH mapping on different days and TME pH quantification. Reproduced with permission from reference (Ma et al., 2018). Copyright 2018, American Chemical Society. **(B)** pH-sensitive fluorescence on-off nanoprobe for FI. Reproduced with permission from reference (Tian et al., 2015). Copyright 2015, Royal Society of Chemistry.

Lower pH in the TME and overexpressed matrix metalloproteases (MMPs) are two indicators of tumor-associated abnormalities. Quantitative and real-time detection of multiple TME factors through non-invasive multimodality imaging is highly informative. Non-invasive visualizations of these abnormality indicators are an effective approach for studying and confirming abnormal tumor signatures *in vivo*. A novel target-triggered dual-ratiometric fluorescent nanoprobe for simultaneous mapping of matrix metalloprotease-9 (MMP-9) activity and extracellular *in vivo* pH has been reported (Ma et al., 2018). The nanoprobe comprises a ratiometric pH-sensitive dye [(*N*-carboxyhexyl derivative of 3-amino-1,2,4-triazole fused 1,8-naphthalimide), ANNA], its fluorescence is quenched while being attached to the surface of an MRI agent (Fe_3_O_4_ NPs) using the peptide substrate of MMP-9, representing the “off” state (Figure 6A). The fluorescence of ANNA was quenched via Förster resonance energy transfer (FRET) between the dye and NPs. However, when the peptide was cleaved by MMP-9, the fluorescence of ANNA was activated (“on” state), allowing for *in vivo* pH mapping. This MMP-9 responsive nanoprobe was also co-labeled with a NIR reference dye (Cy5.5), which serves as an always “on” internal reference dye of the resulting secondary ratiometric fluorescent system. The MMP-9 dependent fluorescence emission from ANNA was compared with that of continually emitting Cy5.5 for quantitatively mapping proteases activity across the entire tumor. Extensive imaging studies using these dual-ratiometric probes in a mouse model of human colon cancer revealed that MMP-9 overexpression and abnormal TME pH have spatio-temporal correlations. Additionally, the synergistic effect of these two characteristics largely controls the heterogeneous invasion of malignant tumors. Another example is the use of the pH-sensitive fluorescence on-off nanoprobe to achieve highly selective tumor imaging (Tian et al., 2015). A pH-activatable fluorophore with an aza-boron-dipyrromethene structure substituted with diethylaminophenyl and bromophenyl groups (NEt_2_Br_2_BDP) and loaded in a nanomicelle composed of a cyclic RGD peptide-poly(ethylene glycol)-block-poly(lactic acid) (cRGD–PEG–PLA) unimers is used as a FI nanoprobe (Figure 6B). The fluorescence was quenched (off) in the nanomicelle at neutral pH (pH-7.4) owing to the photoinduced electron transfer from the diethylaminophenyl moiety to the excited fluorophore. In acidic tumors, the porosity of the nanoprobe increases due to its polymer composition, allowing protons to access the NEt_2_ groups of the loaded NEt_2_Br_2_BDP. This protonation increases the fluorescence at 925 nm, resulting in an enhanced, pH-dependent NIR fluorescence (on).

## pH-Responsive TPNBS for Image-Guided Therapy

pH-responsive TPNBS combine the functions of imaging and therapy in a single NP. Their pH-responsiveness selectively and sensitively activates imaging and therapeutic functions in pathological tissues. However, these functions remain inactive in normal tissues. The selective activation of theranostic functions improves the imaging (S/N ratio) and therapeutic efficacy in tumor tissues. Image-guided therapy using TPNBS enables disease detection, monitoring of disease progression, and evaluation of patient response to therapy. ROS-mediated cancer treatment methods, including light-induced PDT (Lucky et al., 2015), US-induced SDT (Qian et al., 2016), and endogenous ROS-induced CDT (Tang et al., 2019), have emerged as potential alternatives to conventional treatment methods such as chemotherapy and surgery, owing to their minimal or non-invasiveness and improved site-specific action. The efficacy of ROS-mediated PDT, SDT, and CDT depends on the ability of the method to produce an elevated level of cytotoxic intracellular ROS. Compared to neutral or less reactive hydrogen peroxide (H_2_O_2_) ROS, singlet oxygen (^1^O_2_), peroxy radicals (•O_2_H), and hydroxyl radicals (•OH) are highly reactive or more cytotoxic to cancer cells. The overproduction of ROS damages lipid bilayers, proteins, and DNA associated with cancer cells and causes cell death (Yao et al., 2005; Trachootham et al., 2009).

### pH-Responsive TPNBS in PDT

In PDT, light excites a photosensitizer from its ground state to a short-lived excited singlet state. The excited photosensitizer either decays back to the ground state by emitting fluorescence or undergoes intersystem crossing to a relatively long-lived triplet excited state with reversed electron spin. In the triplet excited state, the photosensitizer interacts directly with the substrate and cancer cell membrane, leading to proton or electron transfer and the production of radical cations and anions, which further react with oxygen to produce ROS, such as superoxide anion radicals (O${}_{2}^{-}•$), •OH, and H_2_O_2_. Alternatively, a triplet excited photosensitizer can directly transfer its energy to molecular oxygen in its ground state (^3^O_2_) and convert it into powerful non-radical ROS, ^1^O_2_. Therefore, in addition to light and photosensitizers, oxygen is also a key constituent that determines the success of PDT. In poorly oxygenated tumor tissues (tumor hypoxia), the efficacy of PDT is limited (Zhang et al., 2017a). The low depth of penetration of light into the tissue (<1 mm) is another parameter that limits the application of PDT in deep tumors (Lucky et al., 2015). Tumoral pH-responsive TPNBS were developed to improve the theranostic efficacy of TPNBS for PDT. The pH-responsive TPNBS could augment theranostic efficacy by providing prolonged blood circulation with reduced systemic toxicity, effective tumor accumulation, improved tissue penetration, tumor oxygenation, and tumoral pH-sensitive imaging signal and therapeutic efficacy enhancement.

Qi et al. (2019) reported a pH-responsive simultaneous activation of FI and PDT. Fluorescent dye (Cy7.5), labeled as a pH-responsive copolymer, poly(ethylene glycol)-*b*-poly(2-(isopropylamino) ethyl methacrylate) (mPEG-*b*-PDPA- Cy7.5) micelles were encapsulated with a mitochondria-targeted photosensitizer, triphenylphosphonium-conjugated pyropheophorbide-a (TPPa). The resulting NPs were represented as M-TPPa. The fluorescent signal and photoactivity of M-TPPa were completely switched off through the hetero-FRET from TPPa to Cy7.5 molecules. Hetero-FRET facilitated an amplified photodynamic effect in the tumor tissues and suppressed systemic adverse effects to normal tissues. M-TPPa is exposed to the acidic intracellular environment of human HO8910 ovarian cancer cells. The protonation of the tertiary amino groups of its unimers leads to micelle degradation, followed by the efficient early endosomal escape of TPPa owing to its small molecular structure and high permeability across the lipid membrane. Finally, cationic TPPa relocates into the mitochondria to kill the cancer cells through *in situ* ROS generation upon laser irradiation, providing robust antitumor efficacy with reduced systemic adverse effects. M-TPPa exhibited a 111-fold increase in the fluorescent signal and a 151-fold enhancement in singlet oxygen generation in acidic cancer cells.

**FIGURE 7:**
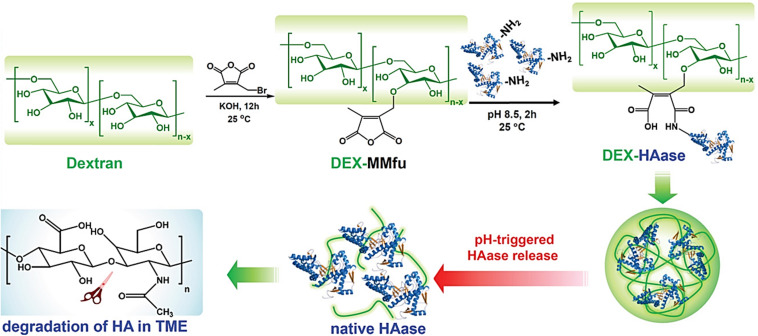
pH-responsive loosening of the condensed extracellular matrix through enzymatic degradation for enhanced tumor permeability of oxygen and nanotheranostics. Reproduced with permission from reference (Wang et al., 2019). Copyright 2019, Wiley-VCH GmbH.

In another study, the enzyme HAase was conjugated to a pH-sensitive traceless linker, 3-(bromomethyl)-4-methyl-2,5-furandione (MMfu), modified dextran (DEX), DEX-MMfu (Figure 7) (Wang et al., 2019). The obtained DEX-HAase NPs, with diminished immunogenicity compared to free HAase, showed enhanced passive tumor (4T1) accumulation owing to its prolonged blood circulation half-life after intravenous injection. The pH-sensitive release of HAase at the TME loosened the condensed extracellular matrix composed of cross-linked HA due to enzymatic degradation, which enhanced the penetration of oxygen and other therapeutic agents (e.g., liposomes). This improved permeability of the tumor, relieved its hypoxia, and enhanced the therapeutic effect of NP-based PDT, followed by the reversal of the immunosuppressive TME to boost cancer immunotherapy. PAI was used to monitor tumor oxygenation (oxygenated hemoglobin). Liposomes loaded with a photosensitizer, chlorine e6 (Ce6), were used as model nanotherapeutics to demonstrate enhanced PDT and immunotherapy as a result of the pH-sensitive augmentation in tumor permeability.

### pH-Responsive TPNBS in SDT

In SDT, US-induced cavitation causes sonoluminescence or pyrolysis, which contributes to the production of ROS from sonosensitizers once it meets oxygen or water at the tumor tissues (Deepagan et al., 2016; Gong et al., 2019). Depending on the frequency, US can be tightly focused, with a penetration of up to several tens of millimeters into soft tissue. Thus, SDT allows access to deeper-seated tumors than PDT (Bailey et al., 2003; Costley et al., 2015). PDT can only be used to treat superficial tumors or endoscopically reachable tumors such as skin cancer, owing to the low penetration depth of light. In PDT treatment, patients must avoid exposure to sunlight after being administered the photosensitizer to avoid light-induced toxicity. Owing to these limitations of PDT, in recent years, research on SDT-based cancer treatment has advanced more rapidly than that on PDT. The lack of an efficient sonosensitizer and poor quantum yield for ROS generation in existing sonosensitizer NPs (Qian et al., 2016) are the primary limitations of SDT. However, these limitations were overcome by developing stimuli-responsive TPNBS (Zhu et al., 2018; Gong et al., 2019). Particularly, a pH-responsive TPNBS for SDT would significantly negate potential side effects in normal tissues and enhance the theranostic efficacy in diseased tissues by providing pH-responsive augmentations in tumor oxygenation, tumor accumulation, tumor penetration, ROS generation, and the S/N ratio of imaging signals.

**FIGURE 8:**
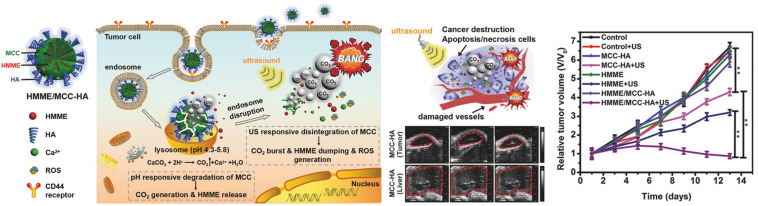
CD44 receptor-mediated endocytosis of HMME/MCC-HA in a cancer cell, followed by the pH/ultrasound dual-responsive decomposition of MCC, CO_2_ bubbling, HMME release, and ROS generation. The CO_2_ burst release causes cancer destruction via cell apoptosis/necrosis and blood vessel destruction. *In vivo* therapeutic efficacy of NPs in MCF-7 tumor-bearing mice confirms effective tumor suppression. Compared to liver lesions with neutral pH, acidic tumor tissues exhibit US contrast enhancement, confirming the pH-responsive augmentation of USI. Reproduced with permission from reference (Feng Q. et al., 2018). Copyright 2018, Wiley-VCH GmbH.

Feng Q. et al. (2018) reported the generation of mesoporous calcium carbonate (MCC) NPs for USI-guided cancer cell necrosis and apoptosis. MCC NPs were loaded with a sonosensitizer [hematoporphyrin monomethyl ether (HMME)], and their surface was decorated with CD44 receptors targeting HA. HA also served as the gatekeeper for the loaded HMME (Figure 8). After receptor-mediated endocytosis, the HMME/MCC-HA decomposed instantaneously via the co-stimulation of inherent tumoral acidic pH and US irradiation, followed by simultaneous HMME release and CO_2_ generation. The degradation associated with the increase in osmotic pressure leads to the redistribution of TPNBS from the endosome to the cytoplasm. In addition to the endosomal escape, the bubbling and bursting of CO_2_ under the US stimulus results in cavitation-mediated irreversible cell necrosis as well as the destruction of blood vessels to further obstruct blood supply, enabling a “bystander effect.” The generation of ROS from HMME, triggered by US, causes cell apoptosis via SDT. Therefore, the same pH-responsive TPNBS are used for apoptosis and necrosis and to promote cell injury in a complementary manner. The generated CO_2_ having the echogenic property, is an effective US contrast agent for the identification of cancerous tissues.

Manganese oxide (MnO_x_) NPs can effectively regulate TME oxygenation owing to their hydrogen peroxidase properties and favorable breakup behavior in mildly acidic and H_2_O_2_-rich TMEs (Zhu et al., 2016). Additionally, MnO_x_ can breakup via an antioxidant (GSH) depletion reaction in mildly acidic pH (Deng et al., 2011); both of these pathways release Mn^2+^ ions, which is a T1-MRI agent. The combination of a sonosensitizer and MnOx can afford MRI-guided augmented SDT due to tumor oxygenation at a mildly acidic TME (Zhu et al., 2018). Similarly, via GSH depletion, sonosensitizer-MnO_x_ ensures the MRI-guided augmentation of SDT due to the depletion of ROS scavenging GSH in acidic cancer cells, resulting in the enhancement of intracellular oxidative stress (Lin et al., 2020).

### pH-Responsive TPNBS in CDT

Chemodynamic therapy is a unique method for endogenously generating highly cytotoxic ROS (•OH) from TME-rich neutral ROS (H_2_O_2_) through a transition metal-ion catalyzed Fenton-like reaction (Chen and Schopfer, 1999; Tang et al., 2019). The released transition metal ions [Fe^2+^ (Li W.-P. et al., 2016), Mn^2+^ (Lin et al., 2020), Ti^3+^ (Wang et al., 2020), Cu^+^ (Ma et al., 2019), etc.] from pH-responsive degradable NPs can be used as CDT agents. Most importantly, as CDT involves ROS activation, compared with SDT or PDT, ROS generation via this process is independent of tumor oxygenation. However, novel TPNBS that follow oxygen-dependent CDT via a light-independent PDT mechanism at an acidic tumoral pH have been recently reported (Lu et al., 2020).

**FIGURE 9:**
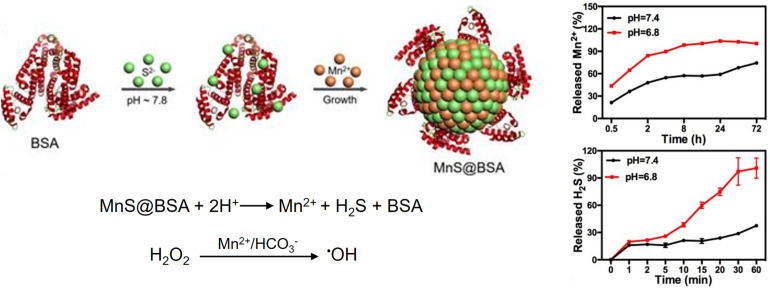
pH-responsive MnS@BSA nanotheranostic for MRI-guided CDT and gas therapy. MnS@BSA enables a pH-responsive release of a CDT agent, Mn^2+^, and H_2_S gas. Reproduced with permission from reference (He et al., 2020). Copyright 2020, Wiley-VCH GmbH.

Tumoral pH-responsive theranostic NPs are being developed for imaging guided CDT. For example, a size-controllable, biodegradable, and metastable γ-phase MnS nanotheranostic (MnS@BSA) was prepared using BSA as a template for tumor pH-responsive *T*_1_-weighted MRI-guided integrated CDT and gas therapy (Figure 9) (He et al., 2020). BSA was used to regulate the size of MnS NPs by tuning the ratio of the proportion of BSA to that of Mn^2+^. In an acidic TME, the as-prepared MnS@BSA underwent pH-responsive dissociation to simultaneously release the MRI or chemodynamic agent (Mn^2+^) and H_2_S gas. The anticancer effect of H_2_S gas, together with the ROS production due to CDT, enables image-guided cancer therapy in 4T1-luciferase mammary tumor xenograft bearing BALB/c mice with significant suppression of tumor growth. Similarly, a pH-activatable chemodynamic system [ultrathin Mn-oxides MnO_x_ nanosheet (Mn^II^)_1_(Mn^III^)_3.4_(Mn^IV^)_2.8_O_11.7_] that can produce ^1^O2 under the trigger of acidity through a novel chemodynamic process has recently been developed (Lu et al., 2020). The ^1^O2 produced from MnO_x_ using this light-free process chemically reacts with thiophene units of semiconducting polymer (SP) NPs (SPNs); thus, the excited SPNs emit near-infrared (NIR) chemiluminescence through a process called chemical electron exchange luminescence (CIEEL). This process further amplifies the yield of ^1^O2 generation. In an acidic tumor environment, the MnOx-SPN system achieved satisfactory chemodynamic therapeutic outcomes *in vitro* and *in vivo* (4T1 female Balb/c mice). The output of ^1^O2 generation was calibrated using ratiometric imaging of chemiluminescence/fluorescence, which provides more accurate *in situ* monitoring of the chemodynamic treatment process.

## Conclusion and Outlook

Owing to their unique physicochemical characteristics, pH-responsive NPs have been extensively investigated for cancer theranostics. The NPs, which exhibit pH-responsive behaviors such as charge conversion, size reduction, and shape changes, would facilitate theranostic efficacy via enhanced tumor accumulation, deep penetration, and/or cellular internalization. In this review, we summarized recent developments that demonstrated how the tumoral pH could be utilized in the design of intelligent NPs with the potential to enhance non-invasive cancer therapy, disease detection/diagnosis, and their combinations.

By utilizing the unique acidic characteristics of tumors, PNBS with pH-responsive amplification of the S/N ratio during tumor-specific imaging has been developed. Thus far, various pH-responsive smart PNBSs have been reported for *in vivo* cancer imaging using suitable techniques such as MRI, USI, PAI, and FI. MRI is a well-established non-invasive whole-body imaging technique owing to the deep tissue penetration of radiofrequency waves. A pH-responsive PNBS for MRI facilitates a tumoral pH-sensitive release of MRI contrast agents, enabling contrast enhancement of deep-seated tumors during diagnosis and treatment. USI is also available for deep-seated tumor imaging owing to the high tissue penetration depth of US. For instance, pH-responsive gas-generating PNBS with strong echogenic signals and excellent echo persistence has been prepared as the potential USI contrast agent. PAI is an emerging hybrid modality that combines optical excitation with ultrasonic detection. It offers deeper tissue penetration and higher spatial resolution than FI, ascribed to US involvement. PAI using a pH-responsive PNBS would allow for semi-quantitative *in vivo* pH sensing and mapping within tumors. Compared to clinically available MRI and USI, the PAI technique would be particularly suitable for *in vivo* pH imaging in small animal models to meet basic and preclinical research needs. *In vivo* applications of FI have limited by the low tissue penetration of light involved, making this approach unsuitable for deep-seated tumor imaging. Similar to PNBS for PAI, fluorescent PNBS are applied to quantitative detection of tumoral pH and tumor imaging in small animal models.

The TPNBS are intelligent, multifunctional nanosystems that combine the imaging and therapy functions into a single NP. Their pH-responsiveness affords selective activation of theranostic functions in pathological tissues, while being inactive in normal tissues. The selective activation of theranostic functions provides improved imaging (S/N ratio) and therapeutic efficacy in acidic tumors with reduced systemic toxicity. Image-guided therapy using TPNBS enables disease detection, monitoring disease progression, and evaluating patients’ responses to treatments. Numerous TPNBS with excellent theranostic functions have been reported recently in basic and preclinical research. These designs need to be relatively simple and biocompatible; most of them, however, are excessively complicated for their clinical translation. Until now, the tumor acidity has been mainly used for developing cancer theranostics. It should be emphasized that the tumor acidity plays a crucial role in tumor initiation, progression, and metastasis. Therefore, developing novel nanotheranostics that modulate tumor acidity would be a promising approach to overcome treatment resistance associated with the current TPNBS.

## Author Contributions

EP, WU, and JP reviewed and evaluated the literature and wrote the manuscript. All the authors contributed to the article and approved the submitted version.

## Conflict of Interest

The authors declare that the research was conducted in the absence of any commercial or financial relationships that could be construed as a potential conflict of interest.

## Abbreviations

PNBS: polymeric nanobiosensors
TPNBS: theranostic polymeric nanobiosensors
TME: tumor microenvironment
ATP: adenosine triphosphate
ROS: reactive oxygen species
RES: reticuloendothelial system
EPR: enhanced permeability and retention
US: ultrasound
PTT: photothermal therapy
PDT: photodynamic therapy
SDT: sonodynamic therapy
CDT: chemodynamic therapy
MRI: magnetic resonance imaging
USI: ultrasound imaging
PAI: photoacoustic imaging
FI: fluorescence imaging
DOX: doxorubicin
HP: tumor-homing peptide
HA: hyaluronan
HAase: hyaluronidase.
